# Ultrastructural visualization of *Orientia tsutsugamushi* in biopsied eschars and monocytes from scrub typhus patients in South Korea

**DOI:** 10.1038/s41598-018-35775-9

**Published:** 2018-11-26

**Authors:** Hyun-Joo Ro, Hayoung Lee, Edmond Changkyun Park, Chang-Seop Lee, Seung Il Kim, Sangmi Jun

**Affiliations:** 1 Drug & Disease Target Team, Korea Basic Science Institute, Cheongju, 28119 South Korea; 2 Center for Convergent Research of Emerging Virus Infection, Korea Research Institute of Chemical Technology, Daejeon, 34114 South Korea; 3 Department of Bio-Analytical Science, University of Science and Technology, Daejeon, 34113 South Korea; 4 Department of Internal Medicine, Chonbuk National University, Jeonju, 54896 South Korea

## Abstract

Scrub typhus, which is caused by *Orientia tsutsugamushi*, is a public health problem in the Asian-Pacific region and is the third most frequently reported infectious disease in South Korea. While ultrastructural studies have been performed on *O*. *tsutsugamushi* in murine fibroblasts, its variable locations in patients have hampered similar studies in humans. Two patients with scrub typhus agreed to provide an eschar biopsy and peripheral blood, respectively. Transmission electron microscopy was performed separately on the necrotic crust and perifocal skin of the eschar, the peripheral blood, and the infected murine L cells. *O*. *tsutsugamushi* was located within or adjacent to the outermost layer of the perifocal inflamed skin of the eschar but not in the necrotic centre. *O*. *tsutsugamushi* in peripheral blood monocytes exhibited the characteristic features of *O*. *tsutsugamushi* in L cells, namely, nearly round shaped bacteria with a size of 1–2 µm and a double membrane bearing a clear halo-like outer layer. The findings confirmed that the bacterium was predominantly located in the inflamed skin around the eschar and that the bacterium had the same ultrastructural features in human monocytes as in L cells. These findings suggest that the perifocal area, not the necrotic centre, should be sampled for diagnosis.

## Introduction

*Orientia tsutsugamushi* is a causative pathogen of scrub typhus. It is an obligate intracellular bacterium that only replicates in the cytoplasm of eukaryotic cells^1^. Scrub typhus is transmitted to humans by bites from trombiculid (chigger) mites. Its symptoms resemble those of dengue and include fever, headache, muscle pain, nausea, and vomiting^1,2^. It can cause multi-organ failure, haemoptysis, and pneumonitis and is fatal in 1.4–6% of cases^2,3^. While it mostly occurs in rural areas, it has been reported throughout the Korean peninsula^4^. At present, there are one million clinical scrub typhus cases every year worldwide^3,5^ and in South Korea, scrub typhus was within the top three of reported cases of category III national notifiable infectious diseases from 2012 to 2018^6,7^. Despite its significance as a public health problem in the Asia-Pacific region, no licensed scrub typhus vaccines are currently available to combat this disease^3^.

An easily detectable diagnostic sign of scrub typhus is the eschar, which forms at the chigger mite bite site^8^. When mature, these lesions consist of an innermost black crust, an erythematous patch in the middle, and a thin surface skin layer that is outlined by white scales^9^. Eschars are detectable within 6–8 days of infection. After maturation, the crust and overlying scales slowly disappear. The eschar is sloughed off approximately 2 weeks after symptom onset^9–11^. While eschars are not always present, 50–93% of patients in South Korea bear them^12^, although this rate does vary slightly depending on the region and the *O*. *tsutsugamushi* strain^4^. Eschar biopsies can be used for PCR-based^13^ and immunohistochemical diagnosis^8,14,15^. Serological tests are also widely used to diagnosis scrub typhus^4^.

Indirect immunofluorescence microscopy studies showed that *O*. *tsutsugamushi* infects^8^ and replicates^16^ in monocytes. Moreover, since the peripheral blood “buffy coat” leukocytes of infected humans still contain *O*. *tsutsugamushi* more than 7 days after infection^17^, these cells may be the site of replication before dissemination^1^. *Ex vivo* experiments also showed that peripheral blood mononuclear cells (PBMCs) and buffy coats from scrub typhus patients contain *O*. *tsutsugamushi*^16^, and *in vitro* experiments showed that it actively infects and replicates in human monocyte-derived dendritic cells^18^.

Many studies have assessed the ultrastructure of *O*. *tsutsugamushi* in cultured L cells, a murine fibroblast line^19,20^. However, the variable location of the bacterium in patients has hampered similar studies in humans. The presence of *O*. *tsutsugamushi* in eschar from patients with scrub typhus has been demonstrated using an immunofluorescence assay and light microscopy with immunohistostaining^8–10,14,15^. Here, transmission electron microscopy (TEM) was performed to observe the ultrastructure of *O*. *tsutsugamushi* in eschars in clinical samples, in peripheral blood from patients, and in L cells infected with a common *O*. *tsutsugamushi* strain (Boryong) in South Korea^4^. Eschars were separated into necrotic crust and perifocal skin to determine the precise distribution of *O*. *tsutsugamushi* in eschars.

## Results

### Evaluation of scrub typhus clinical samples

**Figure 1:**
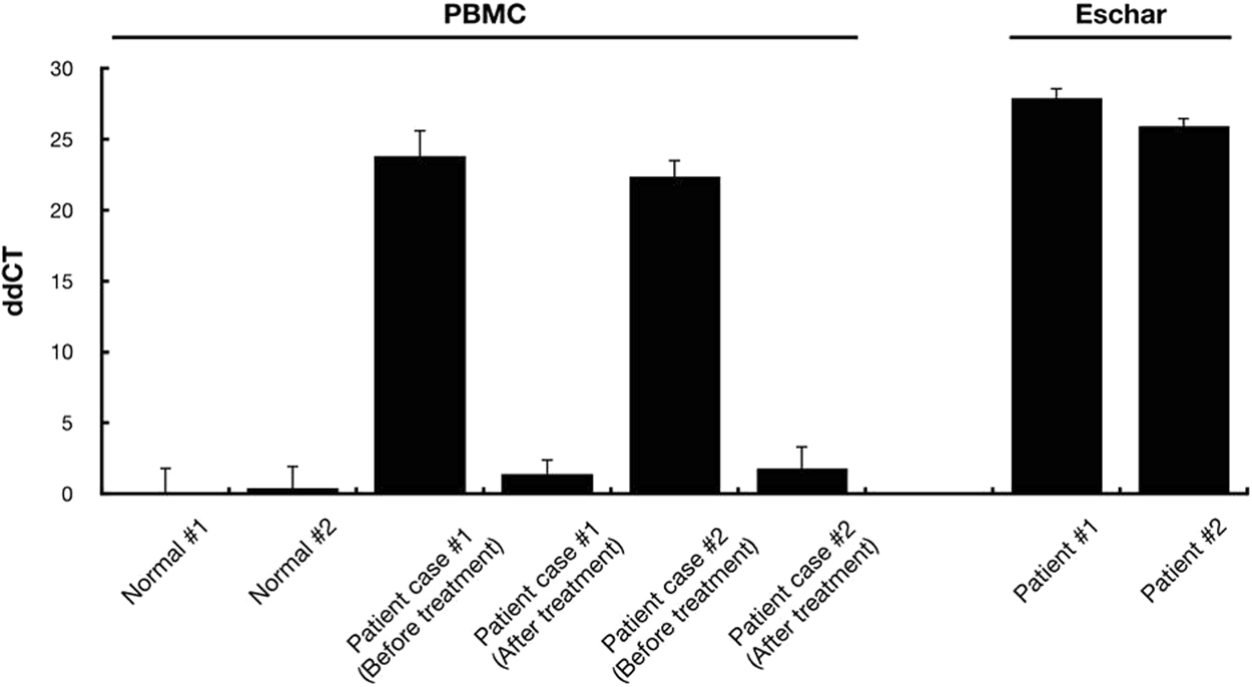
Relative quantification of the *O*. *tsutsugamushi* 56 kDa gene in PBMCs and eschars. The expression level of the 56 kDa gene was evaluated by nested qRT-PCR. The data were normalized to the Gapdh dCT value of the non-infected sample of normal subject #1. The error bars represent the SD of three technical replicates of each sample.

PBMCs in blood and eschars from two scrub typhus patients before and after antibiotic treatment were used for clinical sample evaluation. The samples were tested for the presence of pathogens by using nested qRT-PCR, which showed that the PBMCs and eschars in the samples were positive for a specific scrub typhus gene encoding a 56 kDa protein before antibiotic treatment, thereby confirming the patients were infected with *O*. *tsutsugamushi*. The presence of *O*. *tsutsugamushi* in PBMC was dramatically reduced by antibiotic treatment. The 56 kDa gene was not detected in the normal subjects, which were used as the control (Fig. 1).

### TEM analysis of an eschar

**Figure 2:**
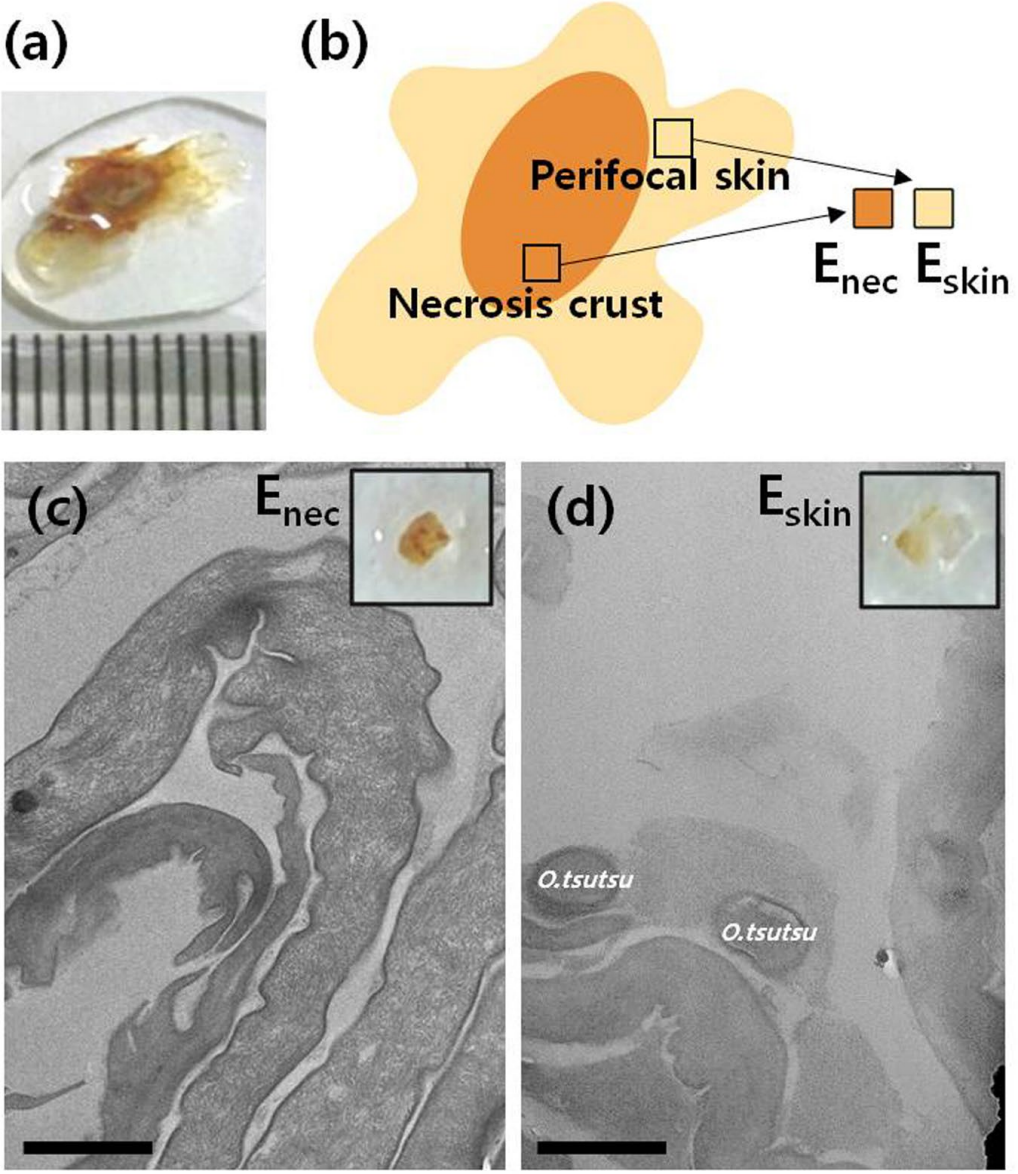
(**a**) Eschar biopsy of a scrub typhus patient (case 1) that was used for transmission electron microscopy (TEM). (**b**) Schematic depiction showing how the eschar biopsy was divided into the central necrotic crust (E_nec_) and the perifocal inflamed skin (E_skin_). TEM micrographs showing that *O*. *tsutsugamushi* (*O*.*tsutsu*) is absent in (**c**) E_nec_ and frequent in (**d**) E_skin_. Scale bars, 1 µm.

A 68-year-old female (case 1) complained of fever, chills, headache, dry tongue, and poor oral intake that began 7 days earlier. Her blood pressure and body temperature were 100/60 mm Hg and 38.9 °C, respectively. Laboratory analyses showed a white blood cell count of 7,810/mL, a platelet count of 111,000/mL, 0.6 mg/dL serum creatinine, 31 IU/L aspartate aminotransferase, 41 IU/L alanine aminotransferase, and 0.81 mg/dL total bilirubin. A positive indirect immunofluorescence assay test and the clinical findings yielded a diagnosis of scrub typhus. A 0.8-cm diameter eschar with a dark-brown crust was found on the right breast. A biopsy that collected the whole eschar and some surrounding inflamed skin was taken (Fig. 2a). On the basis of the appearance and location, the biopsy was divided into the thick innermost dark-brown central crust (E_nec_) and the thin perifocal skin (E_skin_) (Fig. 2b). Both were cut into 1-mm^3^ cubes and sectioned.

**Figure 3:**
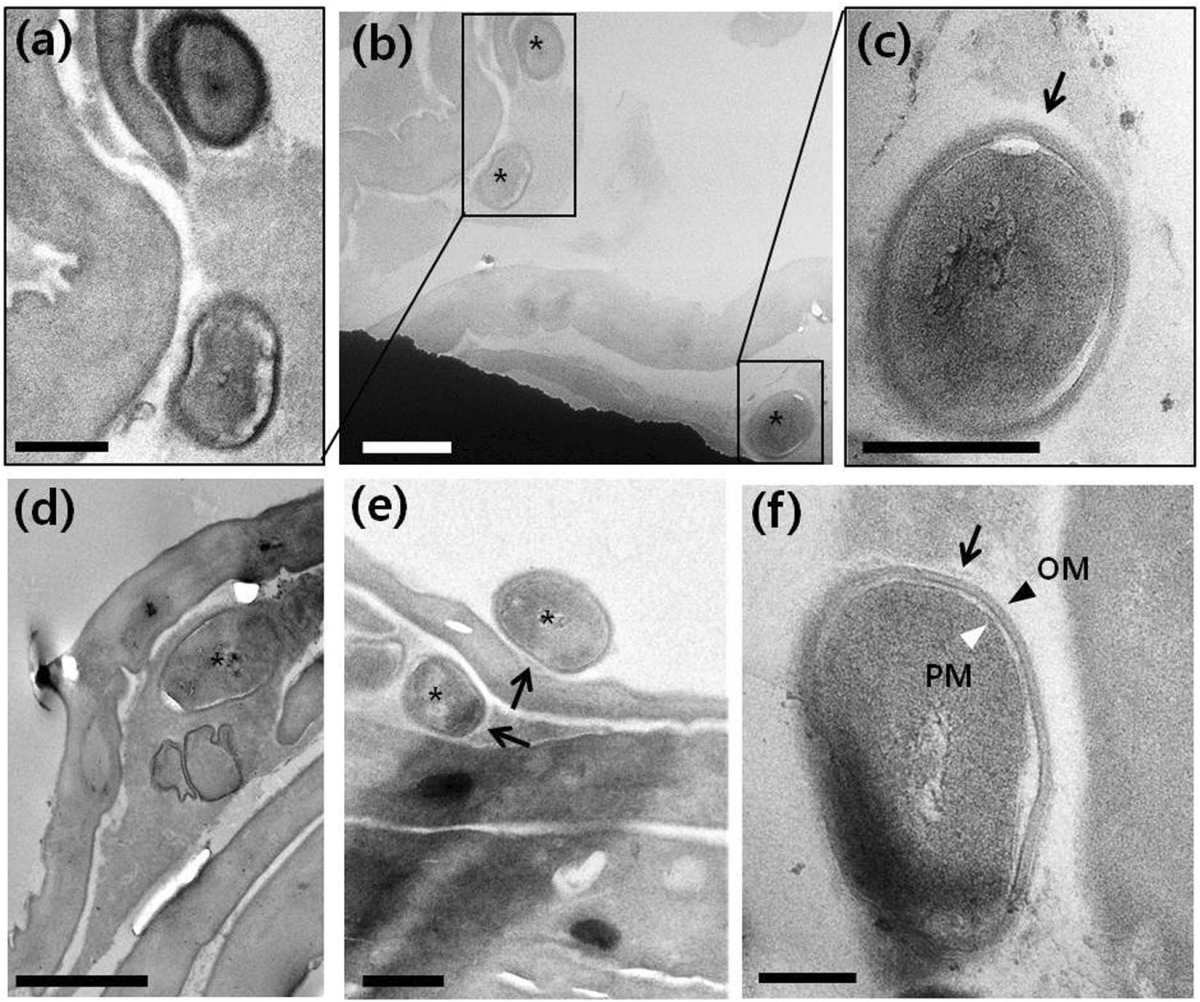
TEM image of scrub typhus patient eschar: (**b**,**d**,**e**) *O*.*tsutsugamushi* in perifocal skin (E_skin_) marked with black asterisks; (**a**,**c**) enlarged image of (**b**,**f**) high magnification TEM image of *O*.*tsutsugamushi* in E_skin_. Black arrows, black arrowhead and white arrowhead point to the electron lucent halo, outer membrane (OM) and plasma membrane (PM), respectively. (**b**,**d**) Scale bar, 1 µm. (**a**,**c**,**e**) Scale bar, 500 nm. (**f**) Scale bar, 200 nm.

To determine the distribution and ultrastucture of *O*. *tsutsugamushi* in eschars, TEM was performed on E_nec_ and E_skin_ (Fig. 2c,d). E_nec_ had a wide intracellular space near the skin surface and more dried dead skin than E_skin_. *O*. *tsutsugamushi* was frequently detected within or adjacent to the skin layers of E_skin_ (Figs 2d and 3 and S1) but was rarely absent in E_nec_ (Figs 2c and S2). We also have performed nested qRT-PCR on two parts, E_nec_ and E_skin_, and confirmed the quantification of bacteria by the location of eschar. Even though a small amount of bacterial DNA could be detected in the necrotic crust from a patient, the nested qRT-PCR results agree with our finding based on the TEM images (Supplementary Fig. S3).

The ultrastructure of *O*. *tsutsugamushi* in E_skin_ is shown in Fig. 3. The bacteria bore the characteristic features of *O*. *tsutsugamushi*, including a nearly round shape with a diameter of ~1 µm^19–22^ and a double membrane with a clear “halo”-like outermost layer^15,19^. The bacteria also had the typical morphology of gram-negative bacteria^23^, including under higher magnification a cell wall, an outer membrane (OM, black arrowhead) associated with an external surface microcapsular layer, an internal plasma membrane (PM, white arrowhead) layer surrounding the cytoplasm, and a periplasmic space that appeared as an electron lucent gap between the outer and cytoplasmic membranes (Fig. 3f). Normally, gram-negative bacteria have a wider periplasmic space than gram-positive bacteria^24^. The peptidoglycan layer was too thin to be resolved in Fig. 3f. A particularly characteristic feature of *O*. *tsutsugamushi* is its outermost electron-lucent halo-like layer adjacent to the cell-wall microcapsule (black arrows in Fig. 3c,e,f), although it is wrapped by an amorphous layer without a clear outline and is not clearly visible around bacteria in *in vitro* infected cells^8,20,21^.

### TEM analysis of *O*. *tsutsugamushi* in a murine fibroblast line and patient monocytes

A 48-year-old female (case 2) visited the emergency room with fever, headache, nausea, and myalgia that started 7 days previously. Her blood pressure and body temperature were 120/86 mm Hg and 38.9 °C, respectively. Laboratory analyses revealed a white blood cell count of 2,630/mL, a platelet count of 106,000/mL, 11.5 g/dL hemoglobin, 0.42 mg/dL serum creatinine, 116 IU/L aspartate aminotransferase, 123 IU/L alanine aminotransferase, and 1.53 mg/dL total bilirubin. An indirect immunofluorescent antibody assay revealed >1:1,520 antibody titers against *O*. *tsutsugamushi*. PBMCs were isolated from blood from the patient. The PBMC sample was somewhat contaminated by red blood cells after isolation. Several studies show that *O*. *tsutsugamushi* infects monocytes^8,16^. Since 10–30% of PBMCs are monocytes^25^, we confirmed *O*. *tsutsugamushi* monocyte infectivity by subjecting the patient’s PBMCs to nested PCR that amplified the gene coding the 56 kDa surface protein of *O*. *tsutsugamushi* (Supplementary Fig. S4).

**Figure 4:**
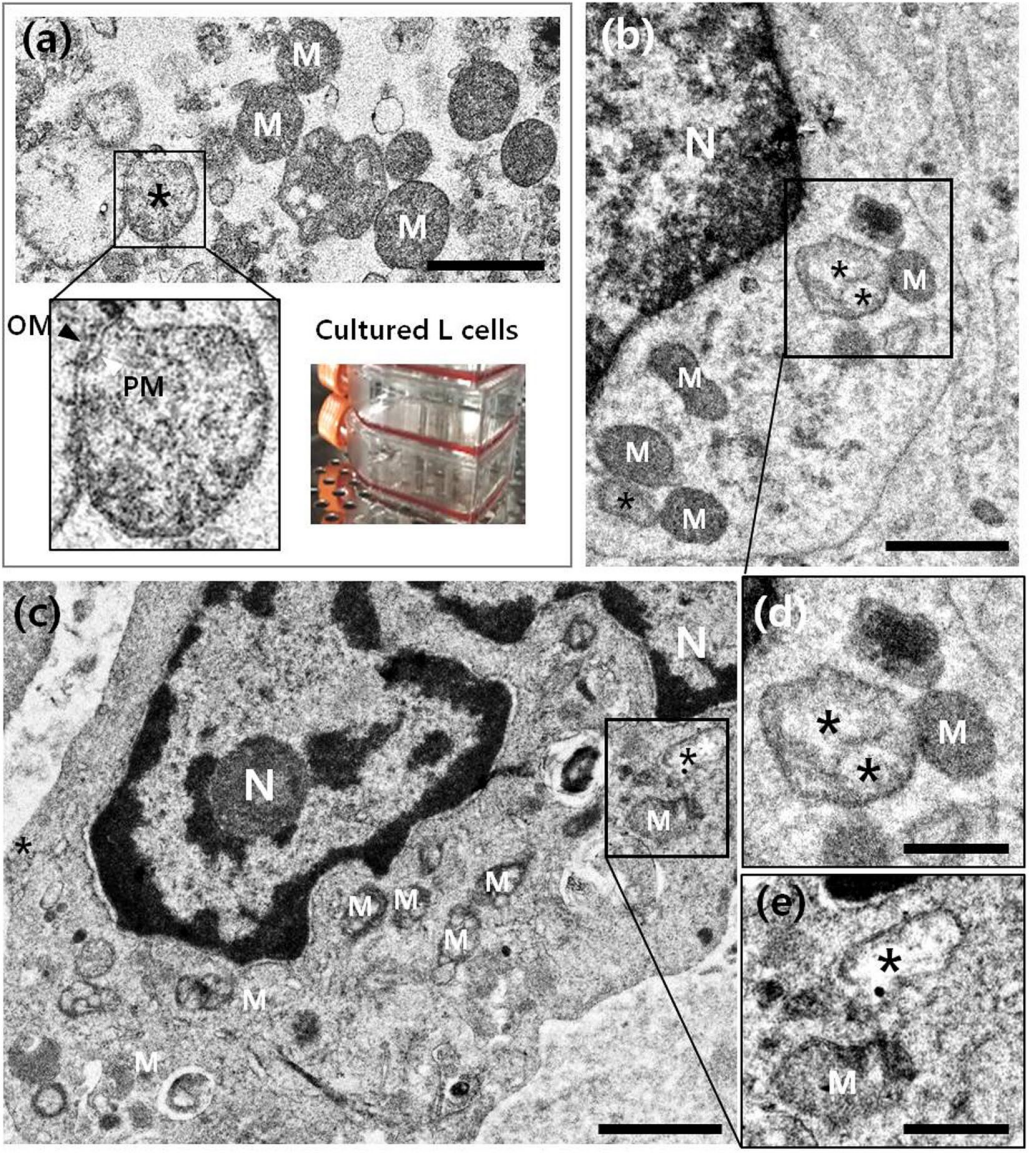
Transmission electron microscope images of *O*. *tsutsugamushi* (black asterisks) in (**a**) cultured murine L cells and (**b**,**c**) peripheral blood mononuclear cells isolated from a scrub typhus patient (case 2). (**d**,**e**) enlarged images of (**b**,**c**), respectively. N: nucleus; M: mitochondria. Scale bars: 1 µm for (**a**–**c**) and 500 nm for (**d**,**e**).

First, we analysed *O*. *tsutsugamushi* in a murine fibroblast cell line to obtain a reference TEM image of the bacteria. We infected L cells with the Boryong *O*. *tsutsugamushi* strain, fixed and sectioned them, and conducted TEM. We readily detected the bacteria in the L cells (black asterisks in Figs 4a and S5): as described previously, the interior of *O*. *tsutsugamushi in vitro* was heterogeneous and granular, the body was nearly round shaped with a size of 1–2 µm and had a double membrane (inset in Fig. 4a)^20,21^. While the double membrane and size and shape of *O*. *tsutsugamushi* showed it resembled mitochondria, the mitochondrial cristae helped to distinguish these organelles from bacteria (Supplementary Fig. S6). At low magnification, the difference in electron density between mitochondria and bacteria helped identify *O*. *tsutsugamushi* (Fig. 4a)^26,27^.

When we subjected the PBMCs from case 2 to TEM, we found *O*. *tsutsugamushi* in monocytes (Figs 4b,c and S7). We confirmed the presence of monocytes in PBMCs according to morphological features, a horse-shoe shaped nucleus including relatively large size^28–30^. The ultrastructural characteristics of the bacteria were similar to those in the infected L cells. They were located in the cytoplasm near the nucleus; the latter bacteria were free of the phagosomal membrane (black asterisks in Fig. 4b,c). Figure 4d,e are enlarged images of the boxed areas in Fig. 4b,c, respectively and show the detailed structure of the bacteria.

## Discussion

*O*.*tsutsugamushi* invades dermal cells and infects antigen-presenting cells in the sub-epidermal zone, including the basal epidermis and superficial dermis^8^. The dry eschar consists of thick layers of compressed epidermis and dermis^15^ with necrosis of the overlying epidermis. Our TEM analysis of an eschar and its surrounding skin showed that *O*. *tsutsugamushi* was detectable within or adjacent to the outermost layers of the perifocal inflamed skin. By contrast, the central necrotic part of the eschar (E_nec_) consisted of dried skin layers only. This is consistent with a previous immunohistochemical phenotyping study that found high *O*. *tsutsugamushi* numbers in the dermo-epidermal junction next to the eschar necrotic zone^8^. In addition, a histopathologic study of eschar and surrounding erythematous lesions showed signs of bacterial infection^14^. Since the eschar is not the main site of *O*. *tsutsugamushi* replication^8^, it is likely that the bacterial load in the eschar reduces over time: since E_nec_ was a mature eschar from a patient whose symptoms started 7 days before biopsy, this may explain why we did not detect *O*. *tsutsugamushi* in E_nec_. Our study contributes significantly to the body of knowledge on the distribution of *O*. *tsutsugamushi* in and around the eschar since previous studies on this issue generally did not specify where in the eschar the bacteria were located. Eschar biopsies are frequently used for diagnosis^8,13^. The fact that *O*. *tsutsugamushi* is mainly located around, and not in, the eschar will improve sampling and diagnosis.

Our TEM analyses showed that *O*. *tsutsugamushi* in the peripheral blood monocytes of a scrub typhus patient were very similar in shape and size to *O*. *tsutsugamushi* in infected L cells. The small bacterial load in human monocytes was addressed in previous studies, which showed that *O*. *tsutsugamushi* replicates more slowly in human monocytes than in L cells^16^, and that monocytes are less frequently infected than dermal dendritic cells in human eschar^8^. We confirmed the infection of PBMCs by nested PCR targeting the 56 kDa gene of *O*. *tsutsugamushi*. The results show that bacteria were present in low amounts in PBMCs and furthermore, the extracellular fraction of bacteria might have influenced the PCR results.

Our TEM analyses of *O*. *tsutsugamushi* in the monocytes of case 2 revealed the probable sequence of events occurring in the early stages of the infection^20,21^, namely attachment of the bacteria to the cell surface, their phagocytosis by the monocyte, their initial localization in tight or loose phagosomes, and then finally, their presence in the cytoplasm near the nucleus and free of phagosomal membranes (Fig. 4b,c)^31,32^. This supports the hypothesis that *O*. *tsutsugamushi* actively escapes from the phagosome to the cytoplasm^33^, after which the naked bacterium moves to the microtubule-organizing centre and replicates itself in the perinuclear region^31^.

Although scrub typhus is a public health problem in the Asia-Pacific region^34,35^, the pathogenic mechanisms of *O*. *tsutsugamushi* and its early dissemination pathways in humans remain poorly understood due to technical difficulties of *in vitro* and *in vivo* studies^1^. Further visual observations of this bacterium in the host will help to confirm, and provide further insights into, its pathogenic mechanisms. This is the first study to provide ultrastructural information about *O*. *tsutsugamushi* in human cells: easier while many studies report the ultrastructure of this bacterium in murine L cells^20,21^, it has been more difficult to acquire ultrastructural images of the infected cells of scrub typhus patients because the location of the bacterium in the human body is so varied and nonspecific^1^.

In summary, our ultrastructural studies showed that while *O*. *tsutsugamushi* is located very rarely in the central necrotic part of the eschar, it is much more prevalent in the perifocal inflamed skin. Moreover, our TEM analyses showed that there were fewer bacteria in the peripheral blood monocytes of a scrub typhus patient than in murine fibroblasts that were infected *in vitro*. We also found several TEM images that directly support previous studies suggesting that *O*. *tsutsugamushi* is first phagocytosed by human monocytes and then released from the phagosome into the cytoplasm, where it then induces its intracellular replication.

## Materials and Methods

### Patients and ethics

Two untreated scrub typhus patients (case 1 and 2) in Chonbuk University Hospital, Jeonju, South Korea, who had a visible eschar, were enrolled in 2016. Both were diagnosed with scrub typhus, as shown by a ≥ four-fold increase in immunofluorescence in the indirect immunofluorescence assay (Green Cross Reference Lab). A skin biopsy that contained the eschar and some surrounding skin was obtained and peripheral blood was collected. Both patients provided informed consent to participate in the study. The study protocol was reviewed and approved by the Institutional Review Board of Chonbuk National University Hospital (IRB Number; CUH 2016–04–007). The study was conducted according to the tenets of the Helsinki Declaration.

### Quantitative real-time PCR (qRT-PCR)

Template DNA was extracted from the PBMCs and eschars using QIAamp DNA Mini Kit (QIAGEN, Venlo, Netherlands) according to the manufacturer’s instructions. The 56 kDa gene of *O*. *tsutsugamushi* was amplified by nested qRT-PCR. Initial rounds of amplifications were performed for 10 cycles using the following primers: 56 kDa-1 forward, 5′-AGCTGATCGTGACTTTGGGATT-3′; 56 kDa-1 reverse, 5′-AGCATTTGATAATGCAGCAAGACC-3′. For the second round of amplification, 1 μl of the PCR product from the initial amplification was used as the template in the qPCR reaction. Nested qRT-PCR was performed using Power SYBR Green Master Mix (Applied Biosystems, Waltham, MA, USA) and the following primers: 56 kDa-2 forward, 5′-CCTAACATACCTCAGGCGCA-3′; 56 kDa-2 reverse, 5′-AACCAAGCGATCCTAGCTGC-3′. Reactions were carried out in 96-well optical PCR plates on an QuantStudio 3 Real-Time PCR Instrument (Applied Biosystems). All samples were run in triplicate and specificity of amplification was verified for each reaction by examination of the corresponding melt curve. The amount of the 56 kDa gene was normalized using the human *Gapdh (*h*Gapdh)* gene and the primers used were as follows: h*Gapdh* forward, 5′-CGGGAAACTGTGGCGTGATG-3′; h*Gapdh* reverse, 5′-ATGACCTTGCCCACAGCCTT-3′.

Delta cycle time (dCT) values were obtained for each reaction by subtracting the CT value of h*Gapdh* in the reaction from the CT value of the tested transcript (56 kDa) run in parallel. Means and standard errors (s.e.m.) were calculated for dCTs obtained from triplicate reactions for each biological sample. To obtain ddCT values, the mean dCT of a given transcript in the wild type was subtracted from the mean dCT of the same transcript from a given experimental sample. Errors in ddCT were propagated from errors of dCT values as the square root of the sum of the squares of the error (s.e.m.) for the dCT value of each transcript.

### Eschar biopsy

The eschar and its inflamed perifocal skin were biopsied when the eschar had a dark-brown crust, and were fixed in 2.5% glutaraldehyde (EMS) and separated into the central necrotic crust and its surrounding skin.

### PBMC isolation from patient blood

Peripheral blood was mixed with an EDTA and D-PBS solution, centrifuged at 700 *g* for 20 min at room temperature, and the PBMC-bearing layer was collected with a Pasteur pipette. The PBMC-bearing layer was washed twice with PBS, mixed with RPMI medium, and centrifuged at 250 *g* for 10 min. The monocytes were isolated manually; some were used for nested PCR and the remainder were fixed in 2.5% glutaraldehyde at 4 °C for TEM.

### L-cell infection with O. tsutsugamushi

L cells were cultured in Dulbecco’s modified Eagle’s medium (HyClone) supplemented with 10% (v/v) fetal bovine serum in a humidified atmosphere with 5% CO_2_ at 37 °C, inoculated with the Boryong *O*. *tsutsugamushi* strain, and cultured for 10 days. After Giemsa staining to confirm infection, the cells were fixed with 2.5% glutaraldehyde in 0.1 M phosphate buffer (pH 7.4), collected by scraping, pelleted by centrifugation at 1000 rpm for 5 min, and fixed in 2.5% glutaraldehyde at 4 °C.

### TEM of an eschar and *O*. *tsutsugamushi*-infected L cells and human PBMCs

The fixed human and murine samples were washed with phosphate buffer, post-fixed with 1% osmium tetroxide (EMS) for 1–2 hours, and dehydrated in a graded ethanol series (50%, 75%, 90%, 95%, and 100%) for 10 min/step. The last step was repeated. The samples were transferred to propylene oxide (EMS) and infiltrated sequentially with 2:1, 1:1, and 1:2 (v/v) propylene oxide: epoxy resin (EMS) for 1 hour/infiltration and then with 100% epoxy resin for 3 hours. For polymerization, the samples were embedded overnight in epoxy resin at 70 °C. Ultrathin plastic sections (80-nm thick) were cut at room temperature by using a Leica EM UC6 ultramicrotome (Leica Microsystems GmbH) and collected on 200-mesh carbon-coated grids. The eschar samples were sectioned parallel to the skin layer. The grids were post-stained with 2% uranyl acetate and 1% lead citrate at RT for 15 and 5 min, respectively. Zeiss LEO912AB 120 kV TEM (Carl Zeiss) and FEI Tecnai F20 200 kV TEM (FEI Company) were used for TEM analysis.

## Electronic supplementary material

Supplementary Information
