# Supplementary material for: Ultrastructural visualization of Orientia tsutsugamushi in biopsied eschars and monocytes from scrub typhus patients in South Korea
Source: Sci Rep. 2018 Nov 26;8:17373. doi: 10.1038/s41598-018-35775-9 (PMC6255831; doi:10.1038/s41598-018-35775-9)
Supplement: Supplementary file 1 — Supplementary Information [file 41598_2018_35775_MOESM1_ESM.pdf]

Supplementary Figures

Ultrastructural visualization of *Orientia tsutsugamushi* in biopsied eschars and monocytes from scrub typhus patients in South Korea

Authors: Hyun-Joo Ro[1,2,3†], Hayoung Lee[1,3†], Edmond C. Park[1,2,3], Chang-Seop Lee[4], Seung Il Kim[1,2,3,*] and Sangmi Jun[1,2,3,*]

† H. J. R. and H. L. contributed equally to this manuscript.

* S. I. K. and S. J. contributed equally to this manuscript.

Affiliations: [1] Drug & Disease Target Team, Korea Basic Science Institute, Cheongju, 28119, South Korea; [2] Center for Convergent Research of Emerging Virus Infection, Korea Research Institute of Chemical Technology, Daejeon, 34114, South Korea; [3] Department of Bio-Analytical Science, University of Science and Technology, Daejeon, 34113, South Korea; and [4] Department of Internal Medicine, Chonbuk National University, Jeonju, 54896, South Korea

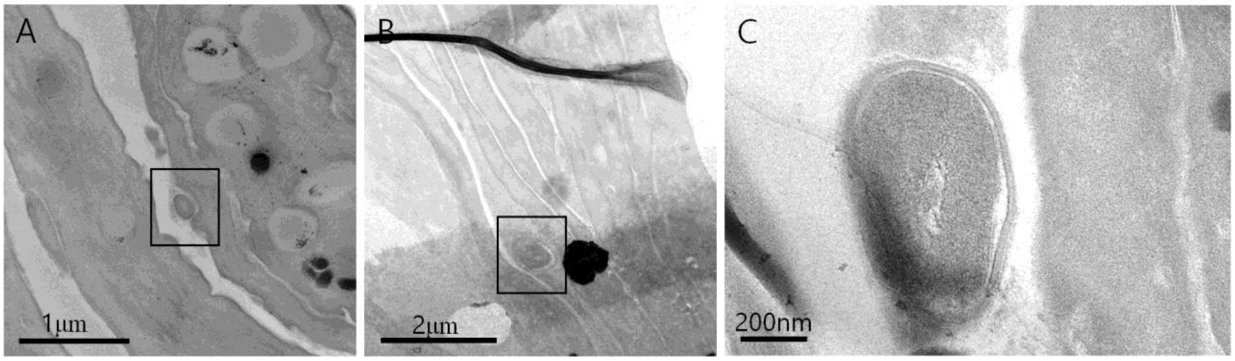

Supplementary Figure S1. (A and B) TEM image showing of *O. tsutsugamushi* (black squares) in E_{skin} of an eschar from a scrub typhus patient. (C) TEM image of the bacterium with high magnification.

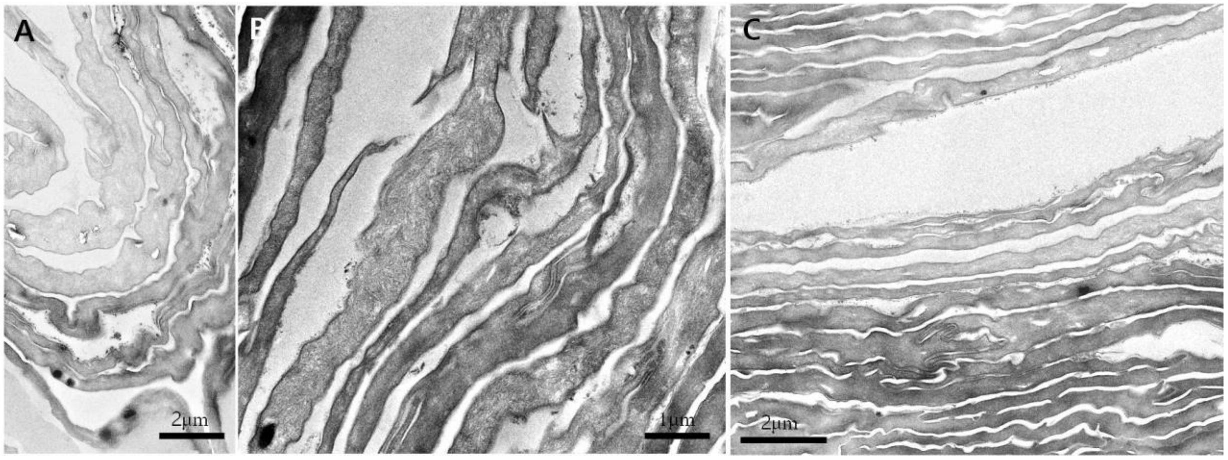

Supplementary Figure S2. TEM images of the central necrotic crust of the eschar (E_{nec}) from case 1. Scale bars: 2 μm in (A and C) and 1 μm in (B).

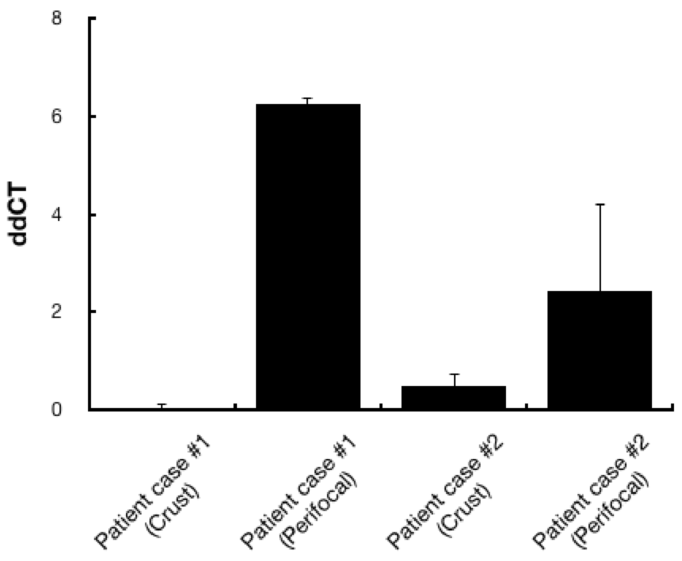

Supplementary Figure S3. Nested qRT-PCR on necrotic crust and perifocal skin of eschars from two patients with scrub typhus, supporting the quantification of bacteria depending on the location of eschar.

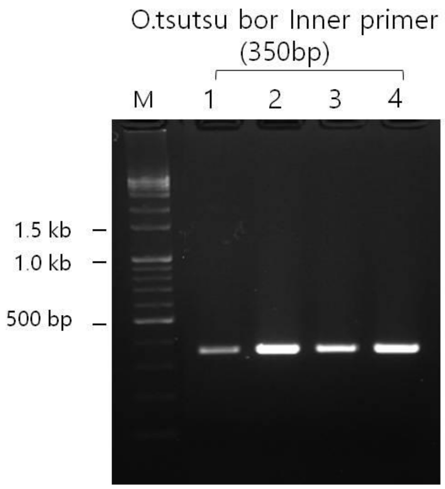

Supplementary Figure S4. Nested PCR targeting *O. tsutsugamuchi* 56 kDa gene from PBMCs of untreated scrub tyuphus patients.

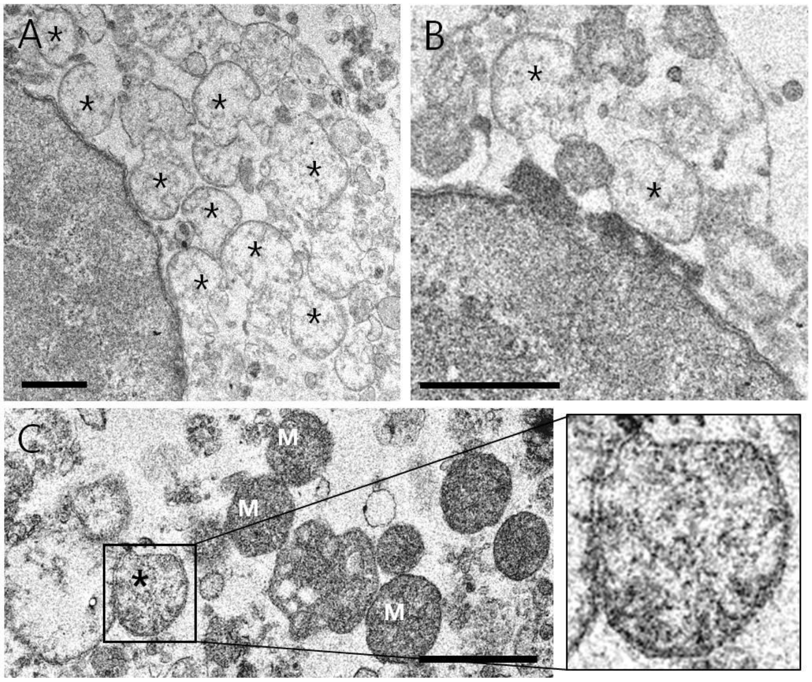

Supplementary Figure S5. (A and B) TEM images of *O. tsutsugamushi* (black asterisks) in cultured L cell inoculated with the Boryong strain. (C) Bacterial double membrane shown by enlarged TEM image (black square). Scale bars, 1 μm.

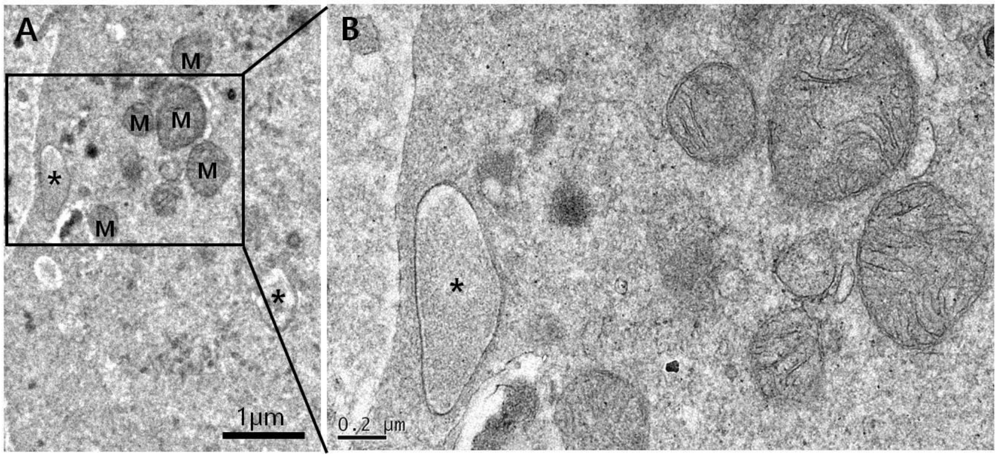

Supplementary Figure S6. (A and B) TEM images of *O. tsutsugamushi* (black asterisk) in the PBMC sample from case 2 and comparison of structural difference between the bacteria and mitochondria. (B) TEM image showing the structure of cristae on mitochondria (M).

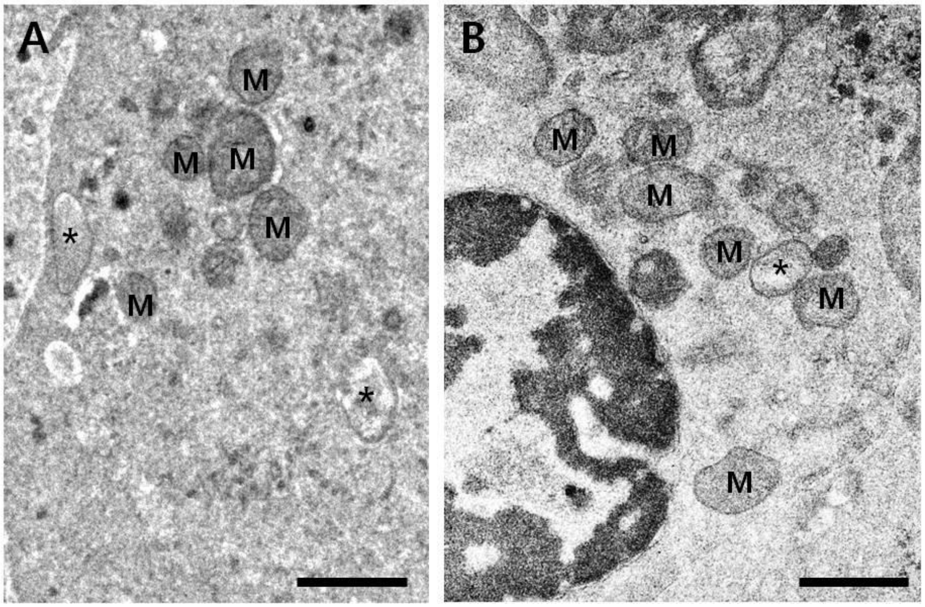

Supplementary Figure S7. TEM images of *O. tsutsugamushi* in the peripheral blood mononuclear cell sample from case 2. (A) *O. tsutsugamushi* (black asterisk) is located separately from the mitochondria (M) in the cytoplasm. (B) *O. tsutsugamushi* in monocytes located between mitochondria. Scale bars, 1 µm.
